# Impact of macroeconomic indicators and regime change on debt stress in Zambia

**DOI:** 10.1371/journal.pone.0311445

**Published:** 2024-10-07

**Authors:** Joseph Phiri, Veronica Chimuka Choolwe, Peter Kondwani Phiri, Michael Chanda Chiseni, Briven Muchanga Simaundu, Evans Osabuohien

**Affiliations:** 1 School of Postgraduate Studies and the Research Unit, University of Lusaka, Lusaka, Zambia; 2 School of Business, University of Lusaka, Lusaka, Zambia; 3 Department of Political Science, Governance and Local Development Unit, Gothenburg University, Göteborg, Sweden; 4 Department of Economics and Development Studies, Covenant University, Ota, Nigeria; 5 DePECOS Institutions and Development Research Centre, Ota, Nigeria; Babes-Bolyai University: Universitatea Babes-Bolyai, ROMANIA

## Abstract

This paper quantifies the economic impact of regime changes and macroeconomic indicators on debt stress in Zambia using the Autoregressive Distributed Lag (ARDL) Bounds test. A 1% short run increase in gross domestic products (GDP) increases debt stress by 3.16% and in the subsequent year lowers it by 7.21%; in the long-run the 1% GDP increases lowers debt stress by 22%. In the long-run, a 1% rise in inflation and the lending rate negatively and positively impacted debt stress levels by -1.52% and 3.90%, respectively. Short-run shocks culminated regime change had short-run adverse impact on debt stress by 3.45% in one year and in the subsequent year by -10.35%, with the variables adjusting to long-run equilibrium at a speed of 71.5%. This is the first paper to quantify the empirical effect of macroeconomic indicators and change in Presidents on debt stress, especially in Africa were the problem of the debt trap is perpetuated. The results from the study implies that to deescalate the impact of debt stress on the economy, the electorate should vote in governments that will not fall short on growth driven macroeconomic policies, making it possible for economic sustainability to prevail; and paper seeks to promote good governance and good economic policies as a premise for sustained macroeconomic stability and development.

## 1. Introduction

Acquiring debt is desirable for countries as it provides the government with the necessary financing that is vital for the running of the country [1]. Recent studies have highlighted that a countries governance system has a significant impact on the amount of public debt and that good governance is of massive importance to the effective management of debt, in some instances concluding that policy changes related to debt repayment enacted by some regimes contributed to political regime change [2, 3]. Other studies have indicated the importance and benefits of a stable macroeconomic environment, which enables debt sustainability and as a consequence contributing to sustained economic growth [4–6]. With a view to financial liability in Zambia, some studies exploring the causal relationship between public debt and economic growth in Zambia concluded that there is a significant relationship between the rate of economic growth and the level of public debt in the country [7, 8], with the latter concluding that there exists a robust and non-linear relationship between sovereign debt and growth in Zambia. This study also concluded that a rising debt to GDP ratio above 40% is negatively associated with economic growth [7].

Prior conducted research studies that have been carried out on this topic have focused on examining the determinants of public debt in Zambia identifying and enumerating factors that have a significant impact on the size of public debt in the country. Some studies have focused on studying the long run relationship between debt and its various determinants whereas others have limited their scope to the short-run [9]. The study by Shiamptanis to investigate the impact of tax austerity on public debt and government debt solvency uses a small open market economy to show that altering the level of taxation can impact the level of debt in the economy, this study highlighted that the level of debt in the country is related to the tax policies in a country, tax austerity affects the position of the effective debt limit [9]. Studies that have focused on the relationship between fiscal policy and government debt have concluded that there is a significant effect of the fiscal factor on the size of public debt of a country, showing that there is a negative significant relationship between government revenue and debt and there is a positive relationship between public expenditure and debt [10, 11].

Additionally, these studies have been carried out at a global or regional level, largely relying on panel data from transitional economies, almost ignoring the behavior of debt and its various determinants in less developed countries [10]. At the time that this paper was written, there were no more than three studies publicized on the impact of macroeconomic variables and regime change on debt stress and the level of debt on the continent, this been the first in Zambia. In June 2023, the Zambian government was able to come to an agreement with its creditors and sealed a $6.3 million debt restructuring deal [12]. This was achieved by the newly elected party that overtook the previously ruling political party in a heavily contested multiparty election race. A similar outcome was achieved was in 2005, under the leadership of Levy Mwanawasa where approximately $4 billion of debt that the country owed to various creditors was cancelled. Even though this was the case, Zambia has experienced an exponential accumulation of debt from the year 2010 to 2020, where debt had rapidly accumulated from 4,252,910,523 in 2010 to 30,045,885,685 in 2020, an unprecedented increase [13]. These events have brought into light matters of the country’s accumulated debt and the relationship of debt stress to the afore mentioned macroeconomic variables including regime change.

In view of the foregoing, this study investigates the relationship between the macroeconomic environment, change in governments and the level of external debt in the Zambian context. The main objective of this paper is to quantify and analyze the economic impact of change in governments and macroeconomic indicators on the magnitude of debt stress in Zambia using the appropriate econometric methods and to offer relevant policy recommendations. The novelty of this paper (in line with the above objective) is been the first paper on the continent and region to econometrically quantify the effect of macroeconomic indicators and change in government on the magnitude of debt stress. As at the year 2020, Zambia’s external debt as % of GDP, lending rate and tax revenue as a % of GDP were pegged at 118%, 10%, and 20%, respectively, while individual and corporate taxes varied as per level of income and circumstances [13]. These statistics underline the importance and timeliness of this study, serving as a model for other developing countries to emulate. This article was test against the following postulated null hypotheses 1 and 2:

Null hypothesis 1: The country’s existing and change in macroeconomic indicators do influence the magnitude of debt stress levels.

Null hypothesis 2: The country’s change in governments do influence the magnitude of debt stress levels.

Against the two hypotheses, the alternative hypothesis will indicate otherwise. This paper is relevant and beneficial to all stakeholders involved in ensuring economic development and in ensuring that the country achieves its vision 2030 in line with Africa’s agenda 2063 as well as the United Nations Sustainable Development Goals (SDGs). The structure of this article will proceed as follows. Section 2 that follows is the data and methods which comprises of the political, economic, and debt history of Zambia, with its latter part consisting of empirical and econometrics procedure used in examining the objectives of the paper. Section 3 are the empirical findings and discussion, and lastly section 4 concludes and makes recommendation on the best economic indicators, governance indicators and policy directives key is deescalating the increasing debt stress levels and their economic impact.

## 2. Data and methodology

### 2.1. Zambian political and economic outlook attributed to debt

A large number of less developed countries have experienced an increase in external debt from 1970 to 1999, this can be attributed to reasons such as consistent budget deficits, a number of global shocks, increased borrowing, and hiked interest rates by western banks to which Zambia owed most of its debt [14]. Many of the least developed countries (LDCs) suffered the debt burden which continued to compound as countries negotiated debt rollovers, rescheduling, or defaults with higher interest rates or lack of access to credit [15]. At this time (1970–1999), the Zambian government was compelled to borrow money in order to keep the country’s economy from falling into a recession of which Zambia adopted democratization. With this democracy system came different political and economic cultures which mostly alluded to the immense dependance on external debt [16]. We see this in the different policies implemented by the United National Independence Party (UNIP) led by Zambia’s very first president Dr. Kenneth Kaunda, as well as, the other policies implemented by the second president of the Republic of Zambia Dr. Fredrick Chiluba who led the Movement for Multiparty Democracy (MMD).

At the time that the Zambian government was compelled to borrow money, Copper prices plunged while the prices of oil rapidly increased, this further deteriorated Zambia’s economic status. Over this period, Zambia’s debt increased from $800 million to $3.2 billion, a 300% increase, most of this debt was obtained from western banks [15, 17]. Structural adjustment programs imposed by the International monetary fund (IMF) and the World Bank as condition for aiding Zambia and other similarly indebted countries seemed to only exacerbate the dire economic times. This program required the implementation of market liberalization policies that resulted in the contraction of the Zambian economy in the 1980s and 1990s, consequently impacting the country’s ability to pay back the debt it owed.

By the early 2000s Zambia’s debt had reached an estimate of $7 billion and had proved to be unsustainable in addition to the change of government of which each political party had its own ways to mitigate or accumulate external debt. Accumulating unfavorable economic conditions made it imperative for the Zambian government to seek ways to pay back their debt. The Zambian government was able to accomplish this through a scheme that aimed to help impoverished countries known as the Heavily Indebted Poor Countries (HIPC) initiative. Approximately $4 billion of debt was cancelled at the completion of this initiative in April 2005 under Dr Fredrick Chiluba’s successor Levy Mwanawasa who also belonged to MMD. However, in 2008 came Rupiah Banda after the death of Levy Mwanawasa under the same political party although at the time of his death Zambia in conjunction with the rest of the world entered a financial crisis.

In as much as the Global Financial Crisis had many direct effects on Zambia’s economy they were at least limited due to the fact that Zambia heavily relied on domestic funding whilst having limited external credit. Rupiah Banda served as president of Zambia from 2008 to 2011, leading the country at a time in which the world economy was experiencing a recession. In 2010, a year before power was transferred from the MMD to the Patriotic Front (PF) the country’s external debt was valued at 4,252,910,523. In 2011 the country had its scheduled elections and power was transferred from the MMD to the Patriotic Front (PF) and this brought into power Micheal Chilufya Sata who was the president of Zambia from 2011 until 2014 when Edgar Lungu took over after his demise. In 2015, a year after there was a regime change, the debt in Zambia was 11,778,536,163. Edgar Lungu was the president of Zambia till the remainder of what was supposed to be Micheal Sata’s was completed in 2016. In 2016, the country went to the pol again. The PF won these elections and Edgar Lungu continued to be the president of the country, ruling the country until in 2021 when the nation conducted general elections where the PF lost power to the United Party for National Development (UPND), which is the incumbent ruling party under the leadership of Hakainde Hichilema. The Table 1 that follows shows selected macroeconomic indicators performance for selected variables external debt stock in USD, per capita GDP, economic growth, inflation and unemployment for selected years between 2010 and 2020.

**Table 1:** Debt stock levels and macroeconomic indicators.

Indicator	2010	2015	2018	2019	2020
External Debt stock (Millions USD)	4,252.91	11,778.53	23,526.27	27,726.25	30,045.88
GDP per capita (USD)	1214.69	1338.29	1368.60	1348.73	1273.87
GDP growth	10.29	2.92	4.03	1.44	-2.78
Inflation	8.50	10.11	7.49	9.15	15.73
Unemployment	13.18	10.12	12.01	12.52	12.84

Source: World Bank (2024)

Sequel to earlier mentions, the external debt stock for Zambia kept increasing from 4 252.91, 11 778. 53, 23 526.27, 27 726.25, and 30 045.88 millions USD for the years 2010, 2015, 2018, 2019, and 2020 respectively which was a total debt rise of 606% over the last decade. Pertaining to per capita GDP for the same respective period 1214.69, 1338.29, 1368.60, 1348.73, and 1273.87 USD culminating into a decade average 1308.84 USD. GDP growth figures 10.30, 2.92, 4.03, 1.44, and -2.79 percentages in the years 2010, 2015, 2018, 2019, and 2020 respectively having recorded a decade growth rate of 3.18%. The inflation rates were 8.5, 10.1, 7.5, 9.2, and 15.8 percentages in the years 2010, 2015, 2018, 2019, and 2020 respectively having recorded a decade average inflation rate of 10.22%. Lastly, the rates of unemployment were 13.19, 10.13, 12.01, 12.52, and 12.85 percentages in the years 2010, 2015, 2018, 2019, and 2020 respectively having recorded a decade average unemployment rate of 12.14%.

### 2.2. Data and econometric procedure of empirical

Data from the World Bank’s World Development Indicators for the period 1990 to 2020 was analyzed annually for entire focus period [13]. The variables of interest included external debt stock % of GDP, real GDP growth constant 2015 USD, inflation rate using the Consumer Price Index (CPI), FDI inflows % of GDP, Tax rate, and lending rates [12]. A detailed description of all the variables used is available in S1 Appendix of this article. The Eviews 12 software was used for the data and econometrics analysis.

This paper applied statistical and time-series econometric procedures. The general formulation of the model is indicated in Eq (1).

Debt=f(GDP,Inflation,FDI,Tax,Lend,DUM) (1)

Where: Debt, GDP, Inflation, FDI, Tax, LEND, and Dum are external debt stock % of GDP, real GDP growth, inflation, FDI inflows % of GDP, Tax rate, lending rates, and Dummy (which takes the value one 1 in the years that had regime change present (New President) and their government and subsequent year, after their inauguration and 0 otherwise), respectively. The stochastic form of the model is shown in Eq (2).

$$Debt=a_{0}+a_{1}GDP+a_{2}Inflation+a_{3}FDI+a_{4}Tax+a_{5}LEND+a_{6}Dum+U_{t}$$ (2)

Where a_0_ is Intercept; while a_1,_ a_2,_ a_3,_ a_4,_ a_5,_ and a_6_ represent coefficients for the variables shown in the general formula and U_t_ is the unobserved Stochastic variable (term).

The econometric procedure for paper proceeds herein.

#### 2.2.1. Unit root test

The unit root was checked for in all the variables as part of the first pre-estimation tests [18–22]. This stage is critical because it recognizes that non-stationary data variables with a unit root are less accurate in larger fractions, which can lead to erroneous findings interpretation [19, 22]. The commonly used Augmented Dickey–Fuller (ADF) is utilized to determine whether stationary is present. Because the ADF test may take serial autocorrelation into consideration when determining whether a unit root exists, it is the recommended method [23]. Below is an indication of the ADF’s general form in Eq (3).

$$\Delta Yt=\beta_{1}+\beta_{2}+\delta Y_{t-1}\sum_{i=1}^{m}\alpha \Delta Y_{t-1}+E_{t}$$ (3)

where ΔYt = represents the related variable, β_1_, β_2_, δ, α = represent parameters in the model, t = represents the time trend, Et = Gaussians white nose with zero mean and possible auto correlation represented by time t. For this research, the unit root test was tested with random walk and drift. As a confirmatory to the ADF unit-root tests, the Phillips–Perron (PP) test was conducted [24]. Both tests have the null hypothesis of the unit root indicating non-stationarity with the alternative hypothesis indicating otherwise [18–22]. However, the limitations of the ADF and the PP are the inability to account for shocks and structural breaks in time series data. In such cases, these two tests (the ADF and PP) usually mistake a structural break as a unit root. To address these defects and account for the presence of structural breaks in time series data, the Zivot–Andrew (Z–A) test was used to confirm stationarity test for the results computed by both the ADF and PP tests [25]. This paper computed all the three results of ADF, PP and Z–A tests to have a strong conclusion on the realization of the time-series pre-estimation tests, giving a better gist and the best time series estimation technique, which proved to be the ARDL Bounds test, which follow s in the next sub-section.

#### 2.2.2. ARDL bounds test

Having tested for the existence of a unit root, the levels of integration for the variables of interest where known, which were a combination of both I(0), and I(1), depending on the unit root test applied making the ARDL Bounds Tests appropriate when analyzing variables that have multiple combinations of order of integration [26]. The advantage of the ARDL Bounds Test is that it addresses the limitations of Engle and Granger (1987) [19] and Johansen and Jeselius (1990) [27], which limit the cointegration steps to variables of the same order of integration I(1). The optimal lags for each of the variables were determined, in the case of this dissertation using the Akaike Information Criterion (AIC), which is preferred in less sample sizes (for example 60 years or less) because of its ability to minimize underestimation while recovering the true lag length, giving it an edge over Hana-quinn and Schwartz criterions [18, 20, 21, 28]. The long-run relationships between the variables were tested, including the short-run impact of regime change and macroeconomic policies on debt stress as necessitated by the objectives of the dissertation [18–22]. The model representation for the ARDL is represented in Eq (4).

$$\Delta {Debt}_{t}=\sigma_{0}+\sum_{i=1}^{p}\sigma_{1i}\Delta {Debt}_{t-p}+\sum_{t=1}^{p}\sigma_{2i}\Delta A{GDP}_{t-p}+\sum_{i=0}^{p}\sigma_{3i}\Delta {Inflation}_{t-p}+\sum_{i-0}^{p}\sigma_{4i}\Delta {FDI}_{t-p}+\sum_{I=0}^{P}\sigma_{5i}\Delta {Tax}_{t-p}+\sum_{I=0}^{P}\sigma_{6i}\Delta {LEND}_{t-p}++\sum_{I=0}^{P}\sigma_{7i}\Delta {Dum}_{t-p}++\lambda_{1}{Debt}_{t-p}+\lambda_{2}{GDP}_{t-p}+\lambda_{3}{Inflation}_{t-p}+\lambda_{4}{FDI}_{t-p}+\lambda_{5}{Tax}_{t-p}+\lambda_{6}{LEND}_{t-p}+\lambda_{7}{Dum}_{t-p}+E_{t}$$ (4)

Δ is the difference operator: p denotes lag length; σ_0_ is the constant term; $\sigma_{1i},\sigma_{2i},\sigma_{3i},\sigma_{4i}\sigma_{5i}{,\sigma }_{6i}{,and\;\sigma }_{7i}$ are error correction dynamics; λ_1_, λ_2_, λ_3_, λ_4_, λ_5,_ λ_6,_ and λ_7_ are long-term coefficients; E_t_ is the white noise disturbance term. The F statistic is used for determining and checking for the presence of cointegration amongst the variables, using the ARDL bounds test [26, 29–33]. The null hypothesis indicates the absence of cointegration against the alternative hypothesis which indicates otherwise. Two bounds are used in examining against this step for cointegration, the lower bound and the upper bound. Higher Wald F statistic, greater the higher I(1) and lower I(0) indicates the presence of cointegration amongst the variables, while the existence of a F statistic lower than the lower and higher bound indicates otherwise. The results are inclusive when the F test lies in between the I(0) and I(1) lower and higher bounds respectively [26, 29–33].

The ARDL Bounds test was first attributed to Pesaran et al. (2001) [26], who showed the flows of always estimating regression with levels (ordinary least squares), in a situation where some variables of interest (in a model) exhibit trending, and seemed to having level of integration either I(0), I(1) or a combination of all but I(2), ARDL Bounds test addresses these, a superiority to (Engel) and (Johansen), which limit their combination to a specific level of integration [26, 29–33]. All these attributes have made ARDL bounds test quite popular and used my scholars who had time series data with similar characteristics, in situation were models contained the long run dynamics, as well as the short error correction dynamics with respective error correction (cointegration) equations [26, 29–34]. Studies on similar topic as this article that applied the ARDL Bounds test include but not limited to the following [8].

As earlier indicated, the ARDL Bounds test, developed to address the limitations of traditional regression models and cointegration methods, offers flexibility in situations where variables exhibit different levels of integration (I(0) or I(1), but not I(2)). This approach is particularly advantageous when dealing with trending variables in time series data, as it allows for the estimation of both short-run error correction dynamics and long-run equilibrium relationships. The ARDL model’s ability to accommodate a mix of integrated variables and provide robust cointegration results without the need for pre-testing of unit roots makes it superior to earlier models like Engle-Granger and Johansen, which require all variables to be of the same order of integration. This has led to the ARDL Bounds test’s widespread adoption in empirical studies that seek to understand complex dynamics in models with varying data characteristics. In the context of this paper, the ARDL Bounds test was timely as it addressed how macro-economic indicators, and change in Presidents impact the country’s debt levels as over the short and long term, knowing that Zambia has experienced the debt problem since independence and this problem might exist for an indefinite time and possibly in perpetuity; and the technique used will capture the problem over short and long spells of time. The next sub-section looks as diagnostic tests used in the analysis.

#### 2.2.3. Post-estimation and stability tests

The estimation model, including its stochastic disturbance term, had to undergo some post-estimation tests to examine the precision of the model. The tests included checking for the presence of autocorrelation in the error term, homoskedasticity (which means constant variance around the error term), ramsey test for non-omitted variable bias or model correct specification, and for normality in the model and residues) [18, 20, 21]. The null hypothesis, which indicates the absence of serial correlation, heteroskedasticity, correct mode specification, and the presence of normality, is desirable [26, 29–33]. The model’s level of stability was also checked using the CUSUM test and the CUSUM squares test was used to verify the absence or presence of (or no structural breaks) having an impact on the model [18, 20, 21, 26]. Other test done were on multicollinearity, that were done through the use of a correlation matrix and variance inflation factor (VIF) [26, 29–32]. All these diagnostic tests are essential, and they reaffirmed the reliability of the model [18, 20, 21, 26]. The following Table 2, that follows shows the descriptive statistics of the raw data used in the empirical analysis.

**Table 2:** Descriptive statistics.

	DEBT	GDP	INFLATION	FDI	TAX	LEND	DUM
Mean	121.1964	4.109990	33.48548	4.816930	15.46037	31.98251	0.322581
Median	129.9672	4.650190	17.96779	4.758709	15.37854	28.20917	0.000000
Maximum	233.7322	10.29822	183.3120	9.604383	19.48288	113.3083	1.000000
Minimum	18.14989	-8.625442	6.429397	1.015757	11.68752	9.479167	0.000000
Std. Dev.	77.52018	3.994226	44.59203	2.393640	1.684011	22.21538	0.475191
Skewness	-0.067391	-1.152882	2.340443	0.183477	0.142951	1.676567	0.759072
Kurtosis	1.498269	4.635493	7.503046	2.309126	3.202323	6.843184	1.576190
Jarque-Bera	2.936426	10.32221	54.49299	0.790452	0.153343	33.60086	5.595494
Probability	0.230337	0.005735	0.000000	0.673528	0.926194	0.000000	0.060947
Sum	3757.088	127.4097	1038.050	149.3248	463.8110	991.4578	10.00000
Sum Sq. Dev.	180281.3	478.6153	59653.46	171.8853	82.24086	14805.69	6.774194
Observations	31	31	31	31	30	31	31

Source: Authors’ computations (2024)

The variables of interest recorded the maximum values of debt, GDP growth, inflation, FDI, tax, and lending rates of 233.73%, 10.29%, 183.31%, 9.60%, 19.48%, and 113.30% respectively, in the respective years 1992, 2010, 1993, 1993, 1990 and 1993. Their respective minimum values were 18.41%, -8.62%, 6.42%, 1.01%, 11.68%, and 9.47%, in the years 2008, 1994, 2011, 1991, 2008, and 2020, with the indicators having the means of 121.19%, 4.10%, 33.48%, 4.81%, 15.46%, and 31.98%, for debt, GDP growth, inflation, FDI, tax, and lending rates respectively. The indicators debt, GDP, FDI, and tax were normally distributed with kurtosis close to or over 3, and skewness near 0, all having respective p-values of over 5%. On the contrary, inflation, and the lending rate were not normally distributed but this did not affect the reliability of the models as all the post-estimation tests of serial-correlation, heteroscedasticity, normality of residues, ramsey test (variable omissions), and stability all came out correct as the results in the next section and part of the appendix indicated. Section 3, results and discussion follows.

## 3. Results and discussion

Table 3 that follows gives results for the pre-estimation tests for the unit-root stationarity test for the variables debt, GDP, inflation, FDI, tax, and lending rates done using the ADF, PP, and Z-A tests.

**Table 3:** Unit root results.

Variable	Test	Level		1^st^ difference	
		Statistic	5% critical	Statistic	5% critical
**Debt**	ADF	-0.368906	-3.562882	-5.046489*	-3.568379
	PP	-0.477722	-3.562882	-5.059422*	-3.568379
	Z-A	-2.328889 (2005)	-4.889812	-6.011055* (2006)	4.859812
**GDP**	ADF	-0.535772	-3.587537	-10.00491*	-3.574244
	PP	-3.482003	-3.568379	-25.57714*	-3.574244
	Z-A	-4.268300* (1998)	-4.859812	-12.14669* (1996)	-4.859812
**Inflation**	ADF	-1.946606	-3.568379	-5.199727*	-3.622033
	PP	-1.332165	-3.568379	-7.342066*	-3.754244
	Z-A	-8.249129* (2002)	-4.859812		
**FDI**	ADF	1.876018	-3.587527	-5.010390*	-3.587527
	PP	-4.057437*	-3.562882		
	Z-A	-4.459192 (1997)	-4.859812	-8.930869* (1996)	-4.859812
**Tax**	ADF	-4.390790*	-3.568379		
	PP	-4.774622*	-3.568379		
	Z-A	-5.194972* (1999)	-4.859812		
**Lend**	ADF	0.772390	-3.622033	-7.194139*	-3.622033
	PP	-4.079368*	-3.568370		
	Z-A	-7.864192* (1993)	-4.859812		

Note: ADF is tested with constant and trend

* Indicates significance at 5% level of significance respectively. The Year of structural break is indicated in brackets for the Z-A test.

Source: Authors’ computation (2024)

In Table 3, the dependent variable debt exhibited a I(1) levels of integration, with statistically significant and higher t-statistics calculated absolute values -5.0464, -5.0594, and -6.0110 for the ADF, PP, and Z-A tests, with the Z-A test break point unit-root test having 2006 as the break year. GDP behaved similarly as was the case with debt with I(1) as the level of integration were the ADF, PP, and Z-A test had statistically significant and higher absolute t-statistics calculated values of -6.0110, -10.0049, and -25.5771, respectively, were the Z-A test had 1996 as the break year. Inflation had a mixture of different levels of integration, with the ADF and PP exhibiting an I(0) levels of statistically significant and higher t-statistics calculated absolute values -5.1997, and -7.3420 for the ADF, and PP, tests, respectively, while its Z-A test had an I(1) level of integration with statistically significant and higher absolute t-statistics calculated values of -8.2491 and having 2006 as the break year. FDI exhibited a mix of I(1), I(0), and I(1) levels of integration for its ADF, PP, and Z-A tests respectively having statistically significant absolute t-statistic calculated values of -5.0103, -4.0574, and -8.9308 respectively with the Z-A test having 1996 as the break year. Tax had all I(1) for the variables levels of integration were the ADF, PP, and Z-A test had statistically significant and higher absolute t-statistics calculated values of -4.3907, -4.7746, and -5.1949, respectively, were the Z-A test had 1999 as the break year. Lastly, the lending rate exhibited a mix of I(1), I(0), and I(0) levels of integration for its ADF, PP, and Z-A tests respectively with statistically significant absolute t-statistic calculated values of -7.1941, -4.0793, and -7.8641 respectively with the Z-A test having 1993 as the break year. The results showed indicated a combination of the I(0) and I(1) levels of integration for all the variables leading to the justification of the use of the ARDL Bounds tests as earlier proposed [26, 29–33]. Table 4 that follows shows the ARDL Bounds tests and the cointegration coefficient results.

**Table 4:** ARDL error correction (with dummy) regression.

Dependent Variable: D(DEBT)				
ECM Regression				
Case 3: Unrestricted Constant and No Trend				
Variable	Coefficient	Std. Error	t-Statistic	Prob.
C	90.78226	12.31808	7.369837	0.0000
D(GDP)	-7.218297	0.976368	-7.393008	0.0000
D(GDP(-1))	3.166889	0.881549	3.592415	0.0022
D(INFLATION)	0.387852	0.132523	2.926676	0.0094
D(INFLATION(-1))	0.391470	0.195754	1.999800	0.0618
D(LEND)	1.337253	0.383094	3.490666	0.0028
D(DUM)	-3.455601	3.451677	-1.001137	0.3308
D(DUM(-1))	-10.35443	3.915412	-2.644531	0.0170
CointEq(-1)*	-0.714534	0.092228	-7.747444	0.0000
R-squared	0.814622	Mean dependent var		-3.588163
Adjusted R-squared	0.744002	S.D. dependent var		23.34443
S.E. of regression	11.81141	Akaike info criterion		8.019335
Sum squared resid	2929.700	Schwarz criterion		8.439694
Log likelihood	-111.2900	Hannan-Quinn criter.		8.153812
F-statistic	11.53524	Durbin-Watson stat		2.113468
Prob(F-statistic)	0.000004			
Test Statistic	Value	Signif.	I(0)	I(1)
F-statistic	9.717991	10%	2.45	3.52
K	4	5%	2.86	4.01
		2.5%	3.25	4.49
		1%	3.74	5.06

Source: Authors’ computation (2024)

As observed in Table 4, GDP growth, inflation, the lending rate as well as regime change had a statistically significant short run effect on debt stress in the county over the focus period. During the short run period, a percentage increase in economic growth reduced debt stress by -7.21% and it the subsequent year increased it by 3.16% respectively. These findings are similar to studies by T.Saungweme(2020) [8] who found that the rate of economic growth influences the level of public debt in Zambia. Debt stress on the other hand increased by 0.38% culminating from a percentage increase in the rate of inflation. The impact of inflation on debt is similar to studies I. S. Mokoginta(2015) [11] Pertaining the rate of lending, a percentage increase in the above culminated into corresponding 1.33% increase in debt stress levels in the county. The findings are in agreement with some studies [35–37]. As far as regime change is concerned, a change in governments culminated into an immediate reduction in debt stress levels by -3.45% in the current year of that change and in the subsequent year a -10.35% reduction. This observation was notable in other studies done [2, 38], and contrasted by a few studies [3].

The impact of the explained variables in debt stress in the short run lasted for a period of 1.4 years as indicated by the error correction term of 0,714 (which was statistically significant), were the effect of all the statistically significant short variables impacted debt stress and converged to long run equilibrium at the speed of 71.5%. This entailed the presence of cointegration amongst the variables as the statistics calculated value had a higher F-statistic 9.717, which was greater than the critical I(0) lower and I(1) upper bounds. The critical lower bound values were 2.45, 2.86, 3.25, and 3.74 at 10%, 5%, 2.5%, and 1% respectively, while the respective I(1) higher critical bound values for the same respective percentages critical values were 3.52, 4.01, 4.49, and 5.06. These results demonstrate cointegration speaking to the justification of the empirical results so far and later, the reason as to why the impact of these indicators on debt stress was examined both in the short run and long run. Table 5, that follows looks at the long run impact of economic growth, inflation, the lending rate, and regime change on debt stress.

**Table 5:** Long run effect of variables on debt stress.

Levels Equation				
Case 3: Unrestricted Constant and No Trend				
Variable	Coefficient	Std. Error	t-Statistic	Prob.
GDP	-21.89646	2.001891	-10.93789	0.0000
INFLATION	-1.525642	0.243409	-6.267808	0.0000
LEND	3.902821	0.426986	9.140393	0.0000
DUM	14.63515	17.67289	0.828113	0.4191

EC = DEBT—(-21.8965*GDP -1.5256*INFLATION + 3.9028*LEND + 14.6352*DUM)

Source: Authors’ computations (2024)

As observed in the above table, a sustained long run percentage increase in GDP growth culminated into decline in debt stress levels by over -21.89%. This observation was similar to related studies [8, 10]. In a similar direction, a long run stable inflation rate increasing by 1% culminated into a decline in debt stress by -1.52%. This observation was similar to related studies [38] and in contrast with a few other studies [10]. Over the long run, both lending rate and regime change positively impacted debt stress, however, inference will not be made on the latter as its coefficient was not statistically significant. Pertaining to the long run effect of lending rate, a percentage increase in the lending rate culminated into a long run increase in debt stress by 3.90%. This observation was similar to related studies [35, 36]. Going back to the overall model, noting Table 3, the model was well-fitted as 81.4% of the model was explained by the regressors as depicted by a higher R-squared of 81.4%. Additionally, the model was well-fitted alluding to a higher F-statistic value of 11.535, which has a corresponding statistical significant p-value (less than 5%). The model was not impacted by serial correlation as Table 4 had a Durbin-Watson stat of 2.113, which is closer to the recommended 2, which entails the absence of serial correlation in the model as a more robust Breusch-Godfrey LM test will verify. The AIC information criteria was used to automatically select the optimal lag of the ARDL Bounds test model and its selected lags 1,1,1,0,0,0,1 debt stock, GDP growth, inflation, FDI, Tax rate, lending rates, and regime change respectively. However, the empirical model only captured statistically significant coefficients in easiness in referencing. Table 6 that follows shows the results for the models post-estimation tests for serial-correlation, heteroscedasticity, and normality tests using the Breusch-Godfrey LM, Breusch-Pagan-Godfrey, and Shapira-Wilk tests respectively.

**Table 6:** Diagnostic tests.

Problem	Test	p-value
**Autocorrelation**	Breusch-Godfrey LM	0.8560
**Heteroskedasticity**	Breusch-Pagan-Godfrey	0.6256
**Model misspecification**	Ramsey RESET Test	0.9577
**Normality**	Shapira-Wilk W	0.700804

Source: Authors’ computations

As noted in the above table, the null-hypothesis for no serial-correlation, homoskedasticity, model misspecification and the presence of normality, which were all desirable, were not rejected with the p–values of 0.8560, 0.6256, 0.9577, and 0.7008, respectively. This showed that the model was good for our analysis and interpretation and the results are good for guiding macroeconomic policies. More additional verification steps were done, with Table 7 below (supported by S2 Appendix), shows the results for the test for collinearity amongst independent variables used in the model.

**Table 7:** Variance inflation factor.

	Coefficient	Uncentered	Centered
Variable	Variance	VIF	VIF
DEBT(-1)	0.026113	43.69218	13.23500
GDP	3.468427	9.997541	4.626696
GDP(-1)	1.610326	4.576009	2.019648
INFLATION	0.029449	7.077507	4.648248
INFLATION(-1)	0.068520	18.70233	11.86265
FDI	5.676679	13.95569	2.785393
TAX	25.08650	503.6385	4.799622
LEND	0.427920	54.71697	18.43103
DUM	72.46447	2.116586	1.386728
C	5479.491	464.1399	NA

Source: Authors’ computations (2024)

The variables of interest as indicated in Table 7 above indicated no concerns of multi-collinearity of regressors are all having a VIF value of less than or centered around the preferred indicator 10, using the centered VIF as shown above. The only VIF indicator that could have had issues for concern is that of the lending rate (18.43); however when confirmed against other tests for multi-collinearity through the use of the correlation matrix, S2 Appendix, were all variables of interest had no collinearity concerns with all notable indicators for all the variable of interest exhibiting values less than 0.80 in absolute terms [18, 20, 21, 26]. S2 Appendix shows confirmation validating the findings in Table 7, which further confirms the complete reliability of the results as confirmed by other tests.

**Fig 1:**
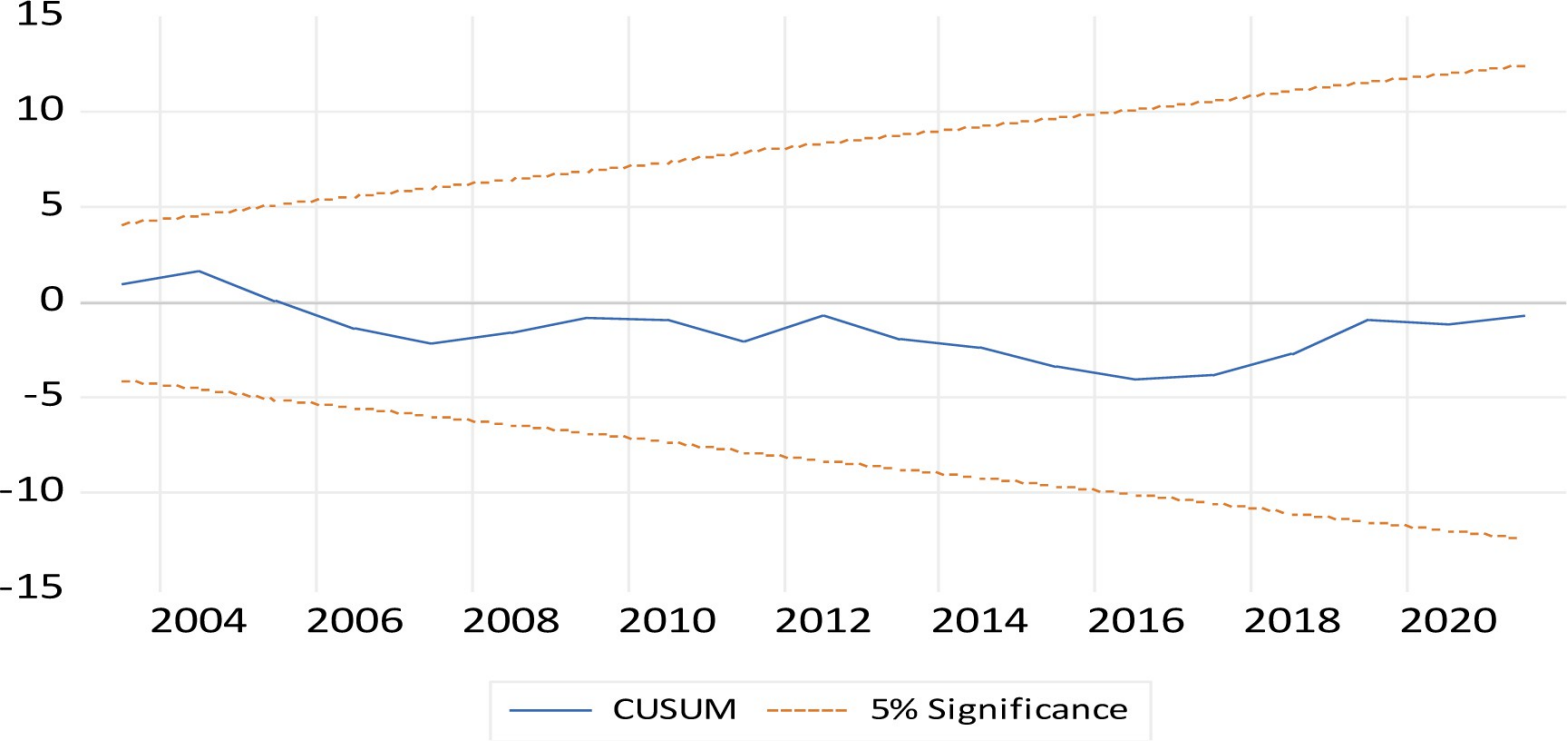
CUSUM test.

**Fig 2:**
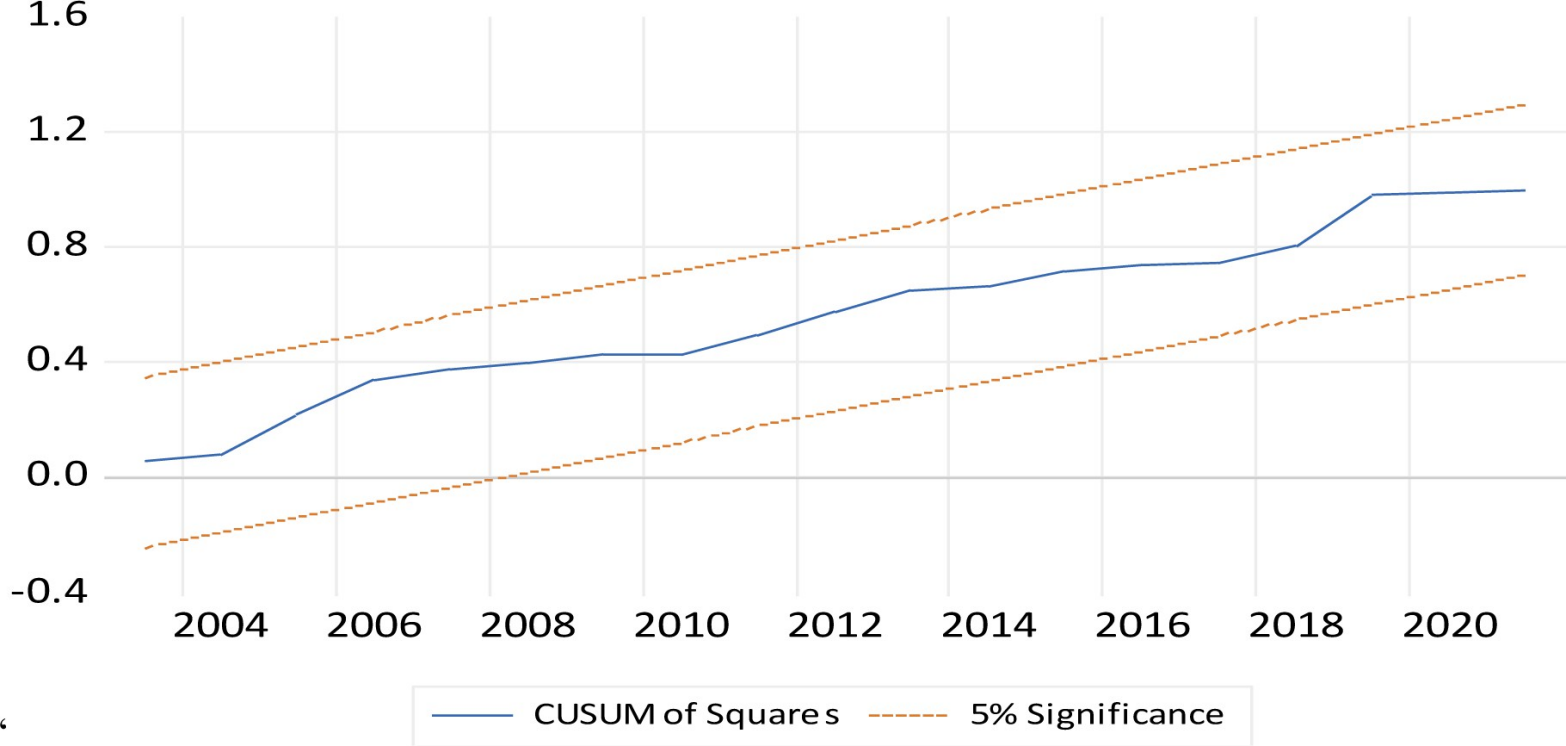
CUSUM squares test.

Fig 1 that follows shows the results of the tests for the stability of the model using the CUSUM test, while the subsequent Fig 2 shows the CUSUM SQUARES test. As shown in Fig 1, the model was stable with its output line within the 10% boundaries as indicated by the dotted line in between the parallel lines in the output figure, which are within 2 standard deviations. The outcome of Fig 1 is similar to that of Fig 2 but the outcome of the latter further enhances the stability of the model as shown in Fig 1 and further verifies that the models was not impacted by any structural breaks and hence Figs 1 and 2, and Table 5 are confirm the reliability of these results discussed.

From the regression analysis carried out it can be seen that regime change had a coefficient of -3.4 in the year in which elections were carried out showing that immediately after regime change debt reduced by 3.4% owing to efforts by the newly elected party to mitigate the debt stress of the country. In the year after elections, the variable regime change had the coefficient -10.35% showing that the new regime reduced debt by 10.35% the year after the change had taken place. The 10% decrease in the level of debt in the country that has occurred in the years after the elections can be attributed to a number of reasons, the most obvious being that immediately after a political party is being that immediately after a political party is elected into power, they initiated negotiations with the various stakeholders, countries, foreign corporations, and other entities who are owed by the country. Additionally, the newly elected political parties pursue policies and programs aimed at addressing the economic status of the country. Examples of programs and policies implemented by newly elected regimes are structural adjustment programs (SAPs) implemented during Dr. Chiluba’s presidency from 1991–2002 to cushion external economic shocks such as changes in global commodity prices and international aid flows [16, 39]. A similar scenario to what led to a decline in the coefficients in the years of change President in which the economy under the Presidency of Mwanawasa instituted reforms that led to debt relief and forgiveness, under the highly indebted poor countries (HIPC) initiative were the country had write off debt in excess of $3.8 billion from its external debt of $6.5 Billion, making its debt roughly 3 billion as at 2008, which made it easier for a macro-economically stable economy in the interim having the exchange rate between 3.5zmw and 4zmw for a dollar and an inflation rate of 12.45%, an improvements from the previous regime of Chiluba making it easier for the then successor to succeed him in an economy with less debt. The subsequent government the PF came it with excessive borrowing in excess of $30 Billion from Chinese debt, Euro bond among others taking advantage of previous cancellations from debt renegotiations from previous regimes [39]. The realizations have indicated governments renegotiations instigated debt reductions thanks to external creditors been favorably to new governments with promise of economic prowess for the citizens, and thanks to the postulation been empirically proven by the short term as indicated in the results presented.

Additionally, renewed confidence placed on the governments of a country after a peacefully successful change of power by investors increases the flows of international funds to the country and makes it easier for the government to receive aid from donors and other international agencies. With regards to the GDP, the results show that in the short run in the year of the regime change a unit increase economic growth represented by GDP reduces the debt stress of the country by 7% and increases it debt stress in the subsequent year by 3%. This relationship between GDP and debt stress in the short run can be as a result of the new agreements that the new regime come to with creditors which could involve taking on more debt in order to control the economic status of the country. In addition to this, at the time of elections, the ruling power is prone to use government funds to carry out their campaign activities which means that they might take on more credit from private creditors and credit institutions. This credit is manifested as the 3% increase in debt in the year.

The long run effect of a unit increase in GDP on the debt stress of the country is that it reduced it by 21%. This can be attributed to the effect of increased productivity on the ability of the country to pay back its debt. From 2010 to 2018, the GDP per capita of the country experienced a steady rise, signifying rising standards of living and increasing capacity of the country (See Table 1). This enables the government to gain increased revenues as they have a higher tax base, making it possible for them to fulfill their credit obligations. The decade had a growth of rate 3.18%, an upward trending economic growth rate owing to the performance of the countries mining sector, including increased exports of agricultural products. These factors can be said to have been augmented by the policy initiatives implemented by the various regimes during the decade.

The lending rate had a long run positive effect on the debt stress, with a percentage increase in the lending rate leading to a 3.9% increase in the level of debt in the country, a reason for this being that when the lending rates increase, creditors tend to demand that the money they are owed be adjusted accordingly. The change in the lending rates impacts the agreements come to between Zambia and the various institutions and entities it owes and if these changes are not accounted for in the negotiations between these parties, renegotiations are initiated to account for the change in the lending rates. A long run controlled inflation had a negative effect on the level of debt of the country with an increase of 1% in the inflation rate leading to a 1.52% decrease in the debt stress faced by the country. The inflation rate signifies the rate at which the prices of goods and services in the country increases and from the findings of this study, a steadily increasing inflation rate enables individuals in an economy to have a more stable real income and a more stable purchasing power enabling the economy to have a population that has enough income to purchase goods, meaning a stable inflation rate contributes to adequate aggregate demand enabling the country’s economy to thrive from all the economic activity that are a consequence of this. The finding of this study have indicated the political and economic landscape of a country has a huge baring on the ability to ability to address the income and debt traps experienced in low income countries and its recommendations serve as a basis for long term macro-economic stability in-line with the eighth development plan of the country, the globe’s vision 2030 and Africa’s agenda 2063.

## 4. Conclusion and recommendations

This study quantified and analyzed the economic impact of changes in governments and macroeconomic indicators on the magnitude of debt stress in Zambia. By applying econometric time-series analysis, including the ARDL Bounds test, the study explored the effects of various macroeconomic variables on debt stress in the country. This study tested against the following two hypotheses: the country’s existing and change in macroeconomic indicators do influence the magnitude of debt stress levels; and that the country’s change in governments do influence the magnitude of debt stress levels. The outcome of the analysis for both hypotheses is in contrast to the null hypothesis and the summary of the results are as such. The findings reveal the complex relationship between economic growth, inflation, lending rates, and debt stress in Zambia. In the short run, a 1% increase in GDP raises debt stress by 3.16%, yet in the subsequent year, it reduces it by 7.21%.

In the long run, a 1% increase in GDP lowers debt stress by 22%, highlighting the importance of fostering economic activities such as investment in agriculture, services, and mining to reduce debt stress. The study found that inflation and lending rates impact debt stress differently. A 1% rise in inflation reduces debt stress by 1.52%, whereas a similar increase in lending rates boosts debt stress by 3.90%. These findings underline the need for prudent monetary policies and effective management of interest rates. The effects of regime changes on debt stress are also significant. Short-term shocks from regime changes negatively impacted debt stress in the first year by 3.45% but improved it by -10.35% in the subsequent year. However, long-term impacts showed an increase in debt stress of 14.63%. This suggests that regime stability is crucial for sustainable debt management, though the impact was significantly pronounced over the short-run period hovering around a year to two of changing Presidents.

Furthermore, the results of this paper provide considerable implications for policy. To reduce the debt stress in Zambia, government can aim to increase the economic growth rate, which can be done through encouraging a majority of Zambian owned business to utilize modern methods of business production and management which are more effective. This can be enabled through state initiated schemes to provide them with training, resource, and finance to institute there business idea. Additionally, the economy can ensure increased investment in agriculture, services, and mining, which will help the economy have less debt stress as this will translate into accelerated economic growth. In addition to the above, lending rates and inflation rates in the country should be monitored and government should aid the various regulatory institutions in ensuring that these do not spiral out of control as these have a bearing on the level of debt in the country. These policies are crucial now more than ever before owing to the fact that the country is experiencing a decline in agriculture and energy production which could have a bearing on economic prospects undermining the nations quest to reduce the debt-GDP ratio. An area of future research includes the impacts of several countries and bilateral debt agreements on the Zambia economy and other regions in SSA including developing countries in different income groups.

## Supporting information

S1 Appendix Detailed definitions of variables used in the paper’s empirical model. (DOCX)

S2 Appendix Correlation matrix for the variables. (DOCX)

S1 File (XLSX)
