# Supplementary material for: Impact of macroeconomic indicators and regime change on debt stress in Zambia
Source: PLoS One. 2024 Oct 7;19(10):e0311445. doi: 10.1371/journal.pone.0311445 (PMC11458046; doi:10.1371/journal.pone.0311445)
Supplement: S1 Appendix — (DOCX) [file pone.0311445.s001.docx]

**Appendix 1:** Detailed definitions of variables used in the paper’s empirical model

| **`Variable** | **Indicator Name** | **Definition and Measurement** |
| --- | --- | --- |
| **Debt** | External debt stocks (% of GNI) | Total external debt stocks to gross national income. Total external debt is debt owed to nonresidents repayable in currency, goods, or services. Total external debt is the sum of public, publicly guaranteed, and private nonguaranteed long-term debt, use of IMF credit, and short-term debt. Short-term debt includes all debt having an original maturity of one year or less and interest in arrears on long-term debt. GNI (formerly GNP) is the sum of value added by all resident producers plus any product taxes (less subsidies) not included in the valuation of output plus net receipts of primary income (compensation of employees and property income) from abroad. |
| **GDP** | GDP growth (annual %) | Annual percentage growth rate of GDP at market prices based on constant local currency. Aggregates are based on constant 2015 prices, expressed in U.S. dollars. GDP is the sum of gross value added by all resident producers in the economy plus any product taxes and minus any subsidies not included in the value of the products. It is calculated without making deductions for depreciation of fabricated assets or for depletion and degradation of natural resources. |
| **Inflation** | Inflation, consumer prices (annual %) | Inflation as measured by the consumer price index reflects the annual percentage change in the cost to the average consumer of acquiring a basket of goods and services that may be fixed or changed at specified intervals, such as yearly. The Laspeyres formula is generally used. |
| **FDI** | Foreign direct investment, net inflows (% of GDP) | Foreign direct investment are the net inflows of investment to acquire a lasting management interest (10 percent or more of voting stock) in an enterprise operating in an economy other than that of the investor. It is the sum of equity capital, reinvestment of earnings, other long-term capital, and short-term capital as shown in the balance of payments. This series shows net inflows (new investment inflows less disinvestment) in the reporting economy from foreign investors, and is divided by GDP. |
| **Tax** | Tax revenue (% of GDP) | Tax revenue refers to compulsory transfers to the central government for public purposes. Certain compulsory transfers such as fines, penalties, and most social security contributions are excluded. Refunds and corrections of erroneously collected tax revenue are treated as negative revenue. |
| **LEND** | Lending interest rate (%) | Lending rate is the bank rate that usually meets the short- and medium-term financing needs of the private sector. This rate is normally differentiated according to creditworthiness of borrowers and objectives of financing. The terms and conditions attached to these rates differ by country; however, limiting their comparability. |
| **Dum** | Categorical dummy 1 representing regime change, 0 otherwise | Regime change in politics is the process of replacing one government regime with another. In think paper regime change refers to change of government, which typically refers to a peaceful transition of leadership within the same political system, often occurring through democratic elections, and does not fundamentally alter the governing framework or constitution (Britannica, 2024). In the context of this paper regime change (represented by a dummy variable) will illustrate the year that we have a change in President including its subsequent year after elections. |

**Note:** The variables used can be found on the listed source below and the data is available open-access

Source: World Banks (2024) World Development Indicators: [World Development Indicators | DataBank (worldbank.org)](https://databank.worldbank.org/source/2?country=IRN&l=en)
