# Supplementary material for: Impact of macroeconomic indicators and regime change on debt stress in Zambia
Source: PLoS One. 2024 Oct 7;19(10):e0311445. doi: 10.1371/journal.pone.0311445 (PMC11458046; doi:10.1371/journal.pone.0311445)
Supplement: S2 Appendix — (DOCX) [file pone.0311445.s002.docx]

**Appendix 2:** Correlation matrix for the variables

|  |  |  |  |  |  |  |  |
| --- | --- | --- | --- | --- | --- | --- | --- |
|  |  |  |  |  |  |  |  |
| Correlation | |  |  |  |  |  |  |
| Probability | DEBT | GDP | INFLATION | FDI | TAX | LEND |  |
| DEBT | 1.000000 |  |  |  |  |  |  |
|  | ----- |  |  |  |  |  |  |
|  |  |  |  |  |  |  |  |
| GDP | -0.628441 | 1.000000 |  |  |  |  |  |
|  | 0.0002 | ----- |  |  |  |  |  |
|  |  |  |  |  |  |  |  |
| INFLATION | 0.659570 | -0.335037 | 1.000000 |  |  |  |  |
|  | 0.0001 | 0.0703 | ----- |  |  |  |  |
|  |  |  |  |  |  |  |  |
| FDI | -0.377951 | 0.610042 | -0.056642 | 1.000000 |  |  |  |
|  | 0.0395 | 0.0003 | 0.7662 | ----- |  |  |  |
|  |  |  |  |  |  |  |  |
| TAX | 0.784287 | -0.715143 | 0.510619 | -0.459605 | 1.000000 |  |  |
|  | 0.0000 | 0.0000 | 0.0039 | 0.0106 | ----- |  |  |
|  |  |  |  |  |  |  |  |
| LEND | 0.670853 | -0.197715 | 0.762513 | 0.052701 | 0.317067 | 1.000000 |  |
|  | 0.0000 | 0.2950 | 0.0000 | 0.7821 | 0.0878 | ----- |  |
|  |  |  |  |  |  |  |  |
|  |  |  |  |  |  |  |  |

Source: Authors’ computations (2024)
